# Label-free mass spectrometry proteome quantification of human embryonic kidney cells following 24 hours of sialic acid overproduction

**DOI:** 10.1186/1477-5956-11-38

**Published:** 2013-08-01

**Authors:** Ville I Parviainen, Sakari Joenväärä, Niina Tohmola, Risto Renkonen

**Affiliations:** 1 Transplantation Laboratory, Haartman Institute, University of Helsinki & HUSLAB, Helsinki University Central Hospital, Helsinki, Finland

**Keywords:** Proteomics, Mass spectrometry, Label-free quantification, Sialic acid

## Abstract

**Background:**

Cell surface glycoprotein sialylation is one of the most ubiquitous glycan modifications found on higher eukaryotes. The surface sialylation pattern of cells is influenced by the cellular environment but also by the Golgi sialyltransferase activity and flux of metabolites through sialic acid producing pathways. Altered cell surface sialic acid patterns have been observed in several cancers and other pathological conditions. In this experiment we examined the cellular proteomic changes that occur in human embryonic kidney cells after 24 hours of sialic acid overproduction using N-Acetylmannosamine. We utilized high resolution mass spectrometry and label free protein quantification to characterize the relative changes in protein abundance as well as multiple reaction monitoring to quantify the cellular sialic acid levels.

**Results:**

Using N-Acetylmannosamine we were able to induce sialic acid production to almost 70-fold compared to non-induced control cells. Mass spectrometric analysis of cellular proteome of control and induced cells identified 1802 proteins of which 105 displayed significant changes in abundance. Functional analysis of the resulting relative changes in protein abundance revealed regulation of several cellular pathways including protein transport, metabolic and signaling pathways and remodeling of epithelial adherens junctions. We also identified several physically interacting co-regulated proteins in the set of changed proteins.

**Conclusions:**

In this experiment we show that increased metabolic flux through sialic acid producing pathway affects the abundance of several protein transport, epithelial adherens junction, signaling and metabolic pathway related proteins.

## Background

**Figure 1:**
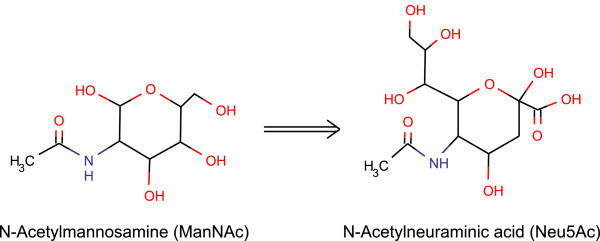
**Chemical structures of ManNAc and Neu5Ac.** Chemical structure representation of N-Acetylmannosamine and N-Acetylneuraminic acid.

Sialic acids are one of the most common terminal monosaccharides found on cell surface glycans of mammals and other higher eukaryotes. Due to its ubiquity and properties sialic acids are involved in many biological functions ranging from early fetal development, cellular recognition and adhesion processes to protein half-life and utilization by influenza virus in entry to cell [1]. The term sialic acid covers more than 50 different glycan structures with a common nine carbon structural backbone and a carboxylic acid group at carbon one. The most common sialic acid structure variant and main metabolic precursor of most other sialic acids is N-Acetylneuraminic acid, Neu5Ac (Figure 1). Cellular Neu5Ac is produced by recycling it from surface glycoproteins using salvage pathway [2] or by de-novo biosynthesis route from other metabolic precursors [3]. The end product of the De novo pathway is activated in the nucleus by N-acylneuraminate cytidylyltransferase to produce CMP-Neu5Ac that can be utilized by the Golgi glycosyltransferases in biosynthesis of glycoconjugates.

Abnormal cell surface sialylation patterns have been described in several malignancies such as colon, breast and brain cancers [4-6]. Traditionally the cause of the aberrant sialylation is thought to originate from defects in Golgi resident glycosyltransferases [7]. However, proteomic studies have demonstrated that changes in metabolic flux through monosaccharide producing pathways can also alter the cell surface presentation of glycoproteins. For example, increase in cellular N-Acetylglucosamine has been shown to influence the branching patterns of surface glycoproteins and also the surface expression of cell growth and differentiation related proteins [8]. Additionally, overproduction of modified sialic acid has been demonstrated to result in an increase in sialylation of only a certain subset of surface glycoproteins [9] rather than the entire glycoproteome.

In recent years mass spectrometry (MS) based proteomics has become a popular method of examining the changes in proteomes in different diseases and cellular states. The increasing resolution and sensitivity of modern mass spectrometers along with advances in sample processing and bioinformatics methods have increased the reliability of MS-based high-throughput analysis in protein quantification. Several methods have been developed to allow reliable identification and quantification of proteins from complex mixtures using isotopically labeled stable compounds [10-13]. Despite being accurate, they suffer from relatively high cost and quality issues due to inefficient labeling and extensive sample handling. More straightforward solution to high-throughput relative protein estimation is label-free MS- quantification. Label-free methods, such as EmPAI [14] and universal signal response factor- based quantification [15] use ion signal intensities acquired by mass spectrometer to assess the amount of peptides within the sample. The area of each ion can be calculated by integrating the extracted ion chromatograms and the relative differences between two samples can then be assessed by comparing the calculated areas of two ions with same mass. Simultaneously the peptide precursor ions are fragmented in the mass spectrometer providing the sequence information required for peptide and protein identification.

In this study we aimed to characterize the functional proteomic changes occurring in human embryonic kidney (HEK293) cells after induction of Neu5Ac overproduction with exogenous N-Acetylmannosamine (ManNAc, Figure 1) [16]. The induction efficiency and Neu5Ac productions was confirmed using multiple reaction monitoring (MRM) mass spectrometry. Protein identification and quantification was performed with ultraperformance liquid chromatography (UPLC) coupled to high performance mass spectrometer using MS^E^ fragmentation and ion mobility separation. Overall we were able to identify 1802 distinct proteins of which 105 proteins showed reliable expression changes. Functional enrichment tools revealed changes in several cellular processes including protein transport, metabolic and signaling pathways and modifications of cellular adherens junctions.

## Results

### Cell cultivation

Cell growth after ManNAc induction was monitored by calculating cells at 0, 6 and 24 hours using hemocytometer. For the first six hours the growth on control and induced samples was identical but after 24 hours the control cells had grown 1.9-fold from time of induction and induced cells 1.6-fold. However, we found no statistical significance (t-test p-value = 0.078) between control and induced cell count. The average viability was 99.2% on control and 98.6% on induced cells.

### Induction of Neu5Ac by MRM

**Figure 2:**
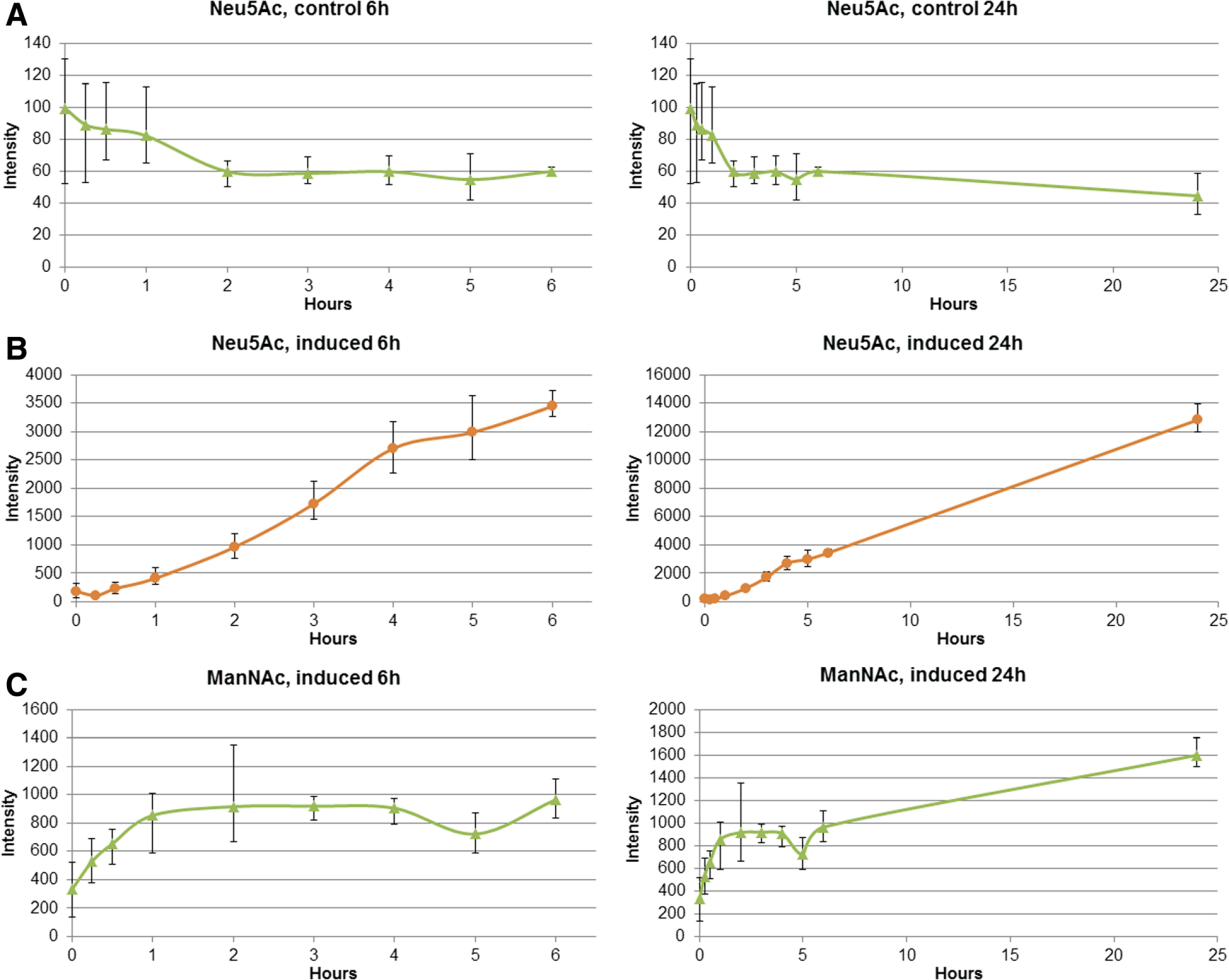
**Neu5Ac and ManNAc quantification with MRM. A**: Neu5Ac amount in control samples without ManNAc induction. **B**: Neu5Ac amount in induced samples with induction using 30 mM ManNAc. **C**: ManNAc amount in induced samples using 30 mM ManNAc. Error bars represent lowest and highest quantification results of the three biological replicates.

The control experiments showed no detectable change in the concentration of Neu5Ac during 24 hours of measurements (Figure 2A). ManNAc- levels were too low on control samples for reliable quantification across all time points. However, the level of Neu5Ac increased extensively in the induced samples (Figure 2B). No rise was seen in the 15 and 30 minute samples but after one hour the levels began to steadily rise. After 24 hours the Neu5Ac level had increased almost 70-fold. The ManNAc level in induced samples began to increase almost immediately after induction but seemed to stabilize after one hour to an average fold increase of 2.7 (Figure 2C). After five hours the levels of ManNAc again began to grow and after 24 hours had increased to about 4.8-fold compared to the original zero hour sample.

### MS data quality evaluation

Before performing the protein expression analysis, the quality of LC-MS^E^ runs was examined. To assess the MS data quality, we used PLGS 3.0 inbuilt data quality tools to examine the technical reproducibility as well as standard error of intensity measurements, retention times and mass errors between MS runs (Additional file 1). In general the mass accuracy of runs, represented as relative standard deviation, was around 1 ppm with error distribution well within acceptable 5 ppm. The average error in EMRT cluster (Exact Mass and Retention Time cluster, denoting the detected and quantified ions) intensities and retention times were 5.1% and 0.8% respectively. Comparison between technical replicate EMRT and protein quantifications exhibited good correlation, as displayed by a 45 degree diagonal intensity distribution between replicates (Additional file 1E). We also compared the repeatability of EMRT and peptide identifications within the technical replicates. On average 32.3% of EMRT clusters and 58.0% of identified peptides were identified in all three technical replicate runs. 55.3% EMRT clusters and 72.4% of peptides were observed in two out of three replicate runs. In general, similar mass, retention time, and intensity errors as well as technical reproducibility were observed in all MS runs validating the data suitability for label free quantification [17-19].

### Quantification results

Overall we were able to identify 1802 distinct proteins with one or more good peptides in the entire data set of control and induced samples (all MS identifications and quantifications in Additional file 2). Of these proteins 1003 could be quantified in all three biological replicates and additional 190 in two out of three biological replicates. 105 proteins passed our criteria for significantly changed proteins (Additional file 3). Interestingly of the 105 proteins only seven showed increase in abundance while 97 seemed to decrease after ManNAc induction.

### Functional annotations

To gain more insight in to biological phenomena occurring after ManNAc induction and overproduction of Neu5Ac we subjected the data to several functional enrichment tools (full details of functional enrichments in Additional file 4).

First, we analyzed the changed 105 proteins using Cytoscape [20] plugin BiNGO [21]. The proteins were enriched to Gene Ontology [22] biological process and cellular component categories. 97 of the 105 proteins could be annotated by BiNGO. In the cellular component category the changed proteins seemed to enrich mainly to plasma membrane (22 out of 97 proteins, P-value = 0.0043) with minor enrichment to extracellular region- category (5/97 proteins, p-value = 0.033) and cytoplasm (78/97 proteins, p-value = 0.049). In biological process category most enriched were protein transport (16/97 proteins, p-value = 0.019) and signal transduction (19/97 proteins, p-value = 0.013) categories.

In order to expand the Gene Ontology enrichments, we next analyzed the data with DAVID Functional annotation Tool (Ver. 6.7) [23,24]. DAVID uses Gene Ontology and other data sources to cluster proteins based on the shared annotations to similarity clusters. These annotation clusters help to visualize the connections shared by different proteins in various categories within Gene Ontology and other annotation sources. The dataset of 105 changed proteins was organized to eleven clusters by DAVID. Largest of the DAVID clusters contained 60 out of the 105 proteins. These included several protein transport and localization categories along with terms associated with G-proteins, GTPase activity, Golgi apparatus and signal transduction.

We also performed pathway analysis to the changed protein set using Ingenuity Pathway Analysis tools (IPA). 52 canonical pathways were enriched in the dataset (p-value ≤0.05). Most notable of these was the Remodeling of epithelial adherens junctions pathway (p-value = 3.02e10^-10^). Several signaling pathways were also altered as well select metabolic pathways including Purine (p-value = 0.000061) and Pyrimidine (p-value = 0.0032) nucleotide biosynthetic pathways and S-adenosyl- L-Methionine biosynthesis pathway (p-value = 0.00011).

**Figure 3:**
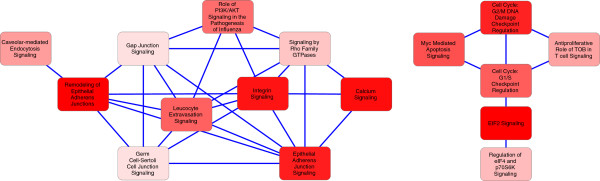
**Interconnected IPA pathways.** Two distinct interconnecting clusters were seen in enriched IPA pathways. One related to signaling pathways and Remodeling of adherens junctions, other related to cell cycle and translation. The connections between nodes represent shared proteins and the color intensity of the nodes reflects the increased enrichment value.

Analysis of pathways that share protein members revealed two distinct interconnected clusters (Figure 3). Larger of these networks contained several signaling networks involved in cellular movement, cell-cell contact and cytoskeletal reorganization. Most of which are also connected to Remodeling of epithelial adherens junction pathway. Second interconnected cluster contained categories on cell cycle, apoptosis and protein synthesis.

### Protein-protein interactions

**Figure 4:**
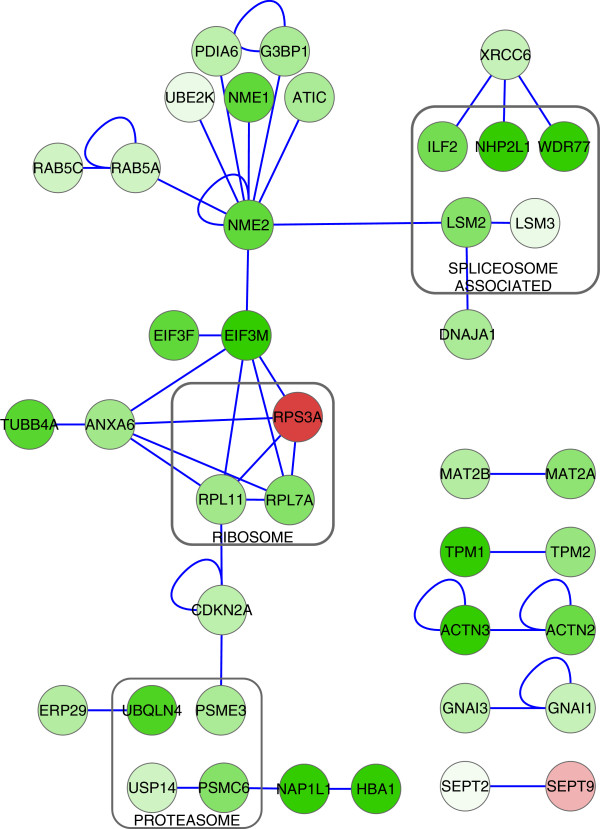
**Physical interactions.** Nine different physically interacting clusters were identified between 40 of the changed proteins. Functional similarities (grey boxes) between interacting proteins were found in proteasomic, ribosomal and spliceosomic proteins. Blue edges represent physical interactions between proteins. Green indicates down-regulation, red up-regulation; more intense color indicates larger change in abundance.

Analysis of the protein-protein interactions between the set of 105 proteins was done by downloading all the known interactions from PINA database [25,26]. This network of 2421 proteins and 4539 interactions was filtered to include only interactions between the changed proteins. 47 interactions were retained between 40 of the 105 proteins (Figure 4).

### WB validation

**Figure 5:**
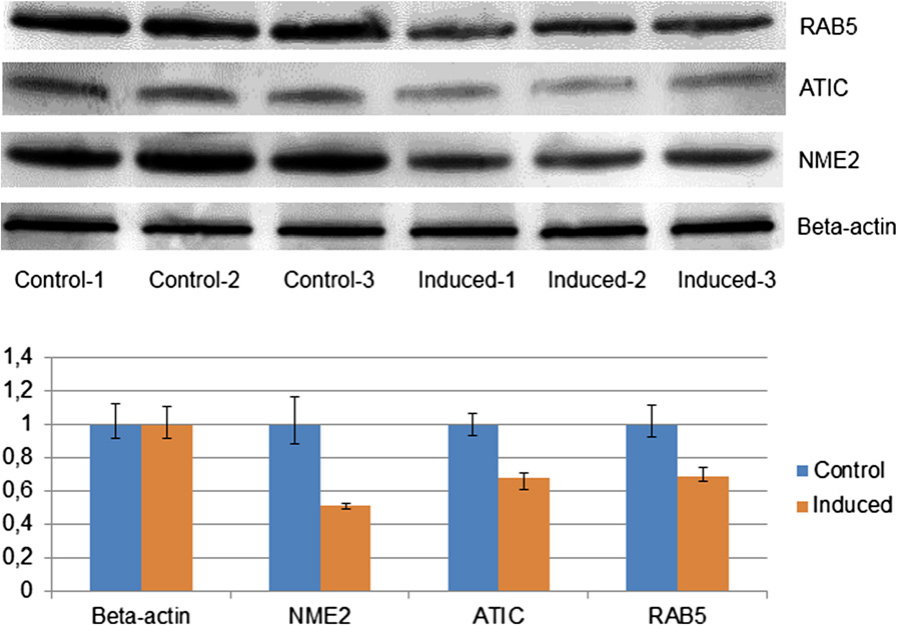
**Western blot validation.** NME2, ATIC and RAB5 were analyzed with Western Blot to validate the MS results. The WB quantification displayed good correlation with MS results. Average NME2 induced/control ratio in MS was 0.55 and with WB 0.69. For ATIC, the ratios were 0.62 (MS) and 0.68 (WB) and for RAB5 the ratios were 0.66 (MS) and 0.69 (WB). Beta-actin was used as loading control. The Western Blot results were normalized to average control values. Bars represent the average of three biological repeats and the error bars represent lowest and highest WB quantification results of the three biological replicates.

To confirm our MS quantification results we assayed the protein abundance changes of three interesting representative proteins, NME2, ATIC and RAB5 by Western Blotting. The results of three biological control and induced replicates (Figure 5) demonstrated similar change in protein abundance in both western blot and MS- quantification. The average induced/control ratios for NME2 were 0.55 with MS and 0.69 with WB, for ATIC the MS quantification ratio was 0.62 and WB 0.68 and with RAB5 MS ratio 0.66 and WB ratio 0.69.

## Discussion

Cell surface sialic acids convey many cellular processes ranging from cell-cell recognition to stabilization of glycoproteins. Abnormal sialylation patterns have been show in many cancer and malignancies. This altered cell surface sialylation is thought to arise from changes in the Golgi resident sialyltransferases but recent experiments have shown that altered flux in the sialic acid producing pathway can also influence the cell surface representation of sialic acids. In the present study we aimed to characterize the proteomic changes that occur after 24 hours of sialic acid, Neu5Ac, overproduction and to elucidate the altered functional processes that are affected by the increase in Neu5Ac production.

Based on previous study [16] we used HEK293 cell line to examine the effect of excess Neu5Ac on the cellular proteome. The production of Neu5Ac was induced by adding 30 mM of N-Acetylmannose to cell culture media. We used high mannose concentration and 24 hours of induction in order to elicit a large production of Neu5Ac and subsequent strong proteomic response in HEK293 cells without significant adverse effects on cell proliferation [16]. Additionally, the long time period of induction should allow an adequate time for proteomic changes to occur and cells to adapt to the increased Neu5Ac concentration.

The ManNAc addition lead to almost immediate increase in cellular ManNAc levels as the concentration of ManNAc began to rise within the first fifteen minutes after induction (Figure 2). However after one hour the ManNAc levels seemed to stabilize to about two to three times higher than in the zero hour sample. This small increase of ManNAc is quite low compared to the excess of added extracellular ManNAc suggesting a regulation of intracellular ManNAc either by reduction of intake from environment or by consumption by ManNAc utilizing enzymes. In contrast to the cellular ManNAc levels, Neu5Ac concentration did not change within the first 30 minutes but began to steadily rise after one hour. The Neu5Ac levels continued to grow linearly for the next 24 hours to almost 70-fold while ManNAc levels increase only 4.8-fold compared to the zero hour samples.

The relative protein quantification analysis identified 105 significantly up- or down-regulated proteins in ManNAc induced cells. Quite surprisingly only seven of the significantly changed proteins showed up-regulation while 100 indicated reduction in abundance. BiNGO analysis of the 105 significantly changed proteins indicated reduction in the abundance Regulation of biological process, Transport, Plasma Membrane and Signal transduction annotated proteins. This suggests that the global adaptation to high ManNAc and Neu5Ac levels is achieved by reducing selected members of cellular transport machinery along with associated plasma membrane and signaling proteins. Such down-regulation could be used to modify the transport to or from plasma membrane in order to reduce the transport of overly sialylated proteins to cell surface or to inhibit the ManNAc transport within the cell.

Further functional analysis of the proteins revealed that the vesicular protein transport was affected by the ManNAc induction and Neu5Ac overproduction (Additional file 3). We identified 18 proteins that were annotated to protein transport categories, most of which were down-regulated. This finding may indicate that the response to high ManNAc and Neu5Ac concentration and possibly to subsequent alterations to cell surface sialylation is regulated by altering the cellular protein transport machinery. Interestingly we identified also eight small GTPases (RAB5A, RAB5C, RAB6B, RAB13, SAR1A, SAR1B, ARF5, and ARF4) in the protein transport- related proteins, seven of which were down- and one up-regulated. Small GTPases are a family of proteins that regulate the targeting and transport on endocytic protein transport vesicles. Small GTPases and their regulating proteins have been shown to confer specify of the cellular transport and also organelle identity [27-29]. Six of identified small GTPases are also annotated to Golgi apparatus. Golgi functions as the main factory of post-translationally modified cell surface proteins and requires several regulating molecules, including small GTPases, to maintain the dynamic transportation routes to and from the apparatus [30]. The protein glycosylation machinery resides in Golgi, so increased sialylation flux within the Golgi and the following regulation of the Golgi transport machinery is not unexpected. It remains unknown whether the reduction of many small GTPases and other transport proteins means reduction on overall transport of cell surface proteins or alterations to specific transport routes within the cellular compartments.

Another major finding in the functional analyses was the reduction in several proteins (RAB5A, RAB5C, DNM2, TUBB1, ACTN2, NME1, TUBB4A, MAPRE1, ACTN3) associated with epithelial adheres junctions. Adherence junctions are plasma membrane bound protein complexes that mediate cellular contacts between cells through sialic acid containing transmembrane glycoproteins, E-cadherins [31-33]. The tightness and stability of adherens junctions between neighboring cells has been shown to be dependent on the cellular density of growing epithelial cell populations [34]. The modifications in junction stability are thought to arise from alterations in E-cadherin glycosylation pattern [31]. The observed changes in the Remodeling of adheres junction pathway proteins after ManNAc induction and Neu5Ac overproduction may be the result of altered sialylation of E-cadherin or other adherens junction proteins. Additionally, the recycling E-cadherin by endosomal transportation route may be altered as we identified E-cadherin recycling related proteins RAB5, DYN2 and NME1 in the set of down-regulated proteins [35,36]. Several signaling pathways that are connected to remodeling of adhesion junctions pathway were also enriched supporting the findings that cell-cell contact points communicate with cellular processes through different signaling routes [37].

The impact of high ManNAc and Neu5Ac overproduction was seen on cellular proliferation level as several interconnected cell cycle, apoptosis and protein translation related signaling pathways were identified in the set of changed proteins (Figure 3). Additionally, ManNAc induction and overproduction of Neu5A affects the metabolic networks for nucleotide production. Purine and pyrimidine biosynthesis pathway proteins, including two committed step catalyzing enzymes of purine biosynthesis pathway (PPAT [38] and ADSS [39]), were reduced after induction with ManNAc. Even though the observed small reduction on cell proliferation was not statistically significant it is possible that the increase in ManNAc and Neu5Ac concentration causes additional strain on the cell population leading to reduced proliferation and modifications to cell growth related signaling processes. Reduced growth in turn leads to reduction in the requirement of nucleotides which is then controlled by proteomic down-regulation of control point proteins of these pathways.

Examination of physical interactions revealed several interacting protein clusters between changed proteins (Figure 4). Among these clusters were several two-protein complexes between protein paralogs, but also three multicomponent clusters with functional similarities between interaction partners. For example, we showed similar down-regulation of interacting proteins MAT2A and MAT2B [40]. S-adenosylmethionine production by MAT2A has been shown to be regulated by association with MAT2B [41]. The association of MAT2A and MAT2B also shields the MAT2A enzyme from proteasomic degradation thus affecting the cellular SAM levels [41]. Additionally, we identified spliceosome- associated (NHP2L1, LSM2, LSM3, WDR77, ILF2), proteasomic (PSMC6 and USP14) and ribosomal (RPL7A, RPL11 and RPS3A) proteins that show changes in abundance. Such alterations to protein members of spliceosome, ribosome and proteasome may indicate regulation of the function or assembly of the respective multiprotein complexes.

## Conclusions

In this experiment we used mass spectrometric label free protein quantification to characterize the relative differences in protein amounts after induction of cellular N-Acetylneuraminic acid overproduction using N-Acetylmannosamine. Functional analysis of significantly changed proteins showed changes in cellular transport- related proteins, remodeling of cell surface adherens junctions and in several signaling and metabolic pathways thus indicating regulation of these processes in response to increased N-Acetylmannosamine and N-Acetylneuraminic acid. Additionally, we identified several co-regulated and physically interacting clusters in the set of significantly changed proteins further elucidating the response to ManNAc induction and excess Neu5Ac.

## Methods

### Cell cultivation

We used mammalian cell line Flip-In −293 (Invitrogen, Carlsbad, CA, USA) transfected with empty expression plasmid. Cells were cultivated in Dulbecco´s Modified Eagle´s Medium with 4.5 g glucose (Lonza, Belgium) supplemented with 2 mM glutamine (Ultraglutamine 1, Lonza), 10% fetal bovine serum (Lonza) and 100 μM Hygromycin (Invitrogen). Cell growth was monitored by calculating cells at 0, 6 and 24 hours after ManNAc induction. All cell calculations were done using Trypan Blue method and hemocytometer. Versene (Gibco Laboratories, Grand Island, NY, USA) was used in detaching cells from cultivation vessels. All experiments were carried out in three individual biological replicates.

### ManNAc induction and cell lysate preparation

Cells were seeded to 6-well plates with 300,000 cells/well in 3 ml media and cultivated for 72 hours at 37°C in 5% CO_2_ atmosphere. Induction with ManNAc (Sigma-Aldrich, St. Louis, MO, USA) was done by adding 300 mM ManNAc in PBS 1:10 to wells yielding final concentration of 30 mM [16]. The control cells were mock-induced with similar volume of PBS. After 24 hours of induction the media was removed and cells were washed three times with 2 ml of ice-cold PBS on ice.

Lysate preparation for Neu5Ac and ManNAc quantification was done by adding 500 μl of ice-cold lysis buffer (50% acetonitrile in H_2_O with 200 μM labeled fructose) to washed cells. Isotopically labeled fructose (Cambridge Isotope Laboratories, Andover, MA, USA) was added to lysis buffer as internal standard to compensate for sample loss during handling. Cells were then allowed to lyse for 10 minutes on ice followed by centrifugation at 16 100 × g at 4°C for 10 min. 400 μl of supernatant was collected and dried using SpeedVac concentrator (Thermo Savant, Holbrook, NY, USA). The dried metabolites were suspended to 0.1% formic acid and filtered using 0.22 μm filters (Millex GW PDVF filter, Millipore, Ireland) and stored at −70°C until MS analysis.

Samples for MS protein quantification were prepared by adding 500 μl of cold lysis buffer (1% SDS in 50 mM ammonium bicarbonate, pH = 8.3) to cells and incubating for 30 minutes on ice. Cell lysate was then collected and snap frozen with liquid nitrogen. Lysates were stored at −70°C until trypsin digestion.

### Trypsin digestion and preparation for LC-MS

Lysates were thawed on ice and then centrifuged 16 100 × g at 4°C for 30 minutes. 400 μl of lysate was collected and protein concentration was assessed using BCA protein assay kit (Thermo/Pierce, Rockford, IL, USA). 25 μg of protein was used in digestion. The volumes of each sample was set to 60 μl with LC-MS lysis buffer and reduced with 5 mM dithiotreitol for 45 minutes at 60°C. Samples were cooled to room temperature and alkylated with 12.5 mM iodoacetamide in dark at room temperature. SDS concentration was reduced with 90 μl of 50 mM ammonium bicarbonate before addition of 1.25 μg trypsin (Trypsin Gold, Mass Spectrometry Grade, Promega, Madison, WI, USA). Samples were digested for 18 hours at 37°C. After digestion SDS was removed from samples with Detergent removal spin columns (Thermo/Pierce, Rockford, IL, USA) using modified manufacturer’s protocol [42]. After SDS removal peptides were purified using C18 spin columns (Thermo/Pierce) by manufacturer’s protocol. Peptides were then dried in Speedvac and stored at −70°C until analysis.

### Neu5Ac and ManNAc quantification

Samples for MRM quantification were thawed on ice and 50 μl of sample was analyzed with LC-MS. The liquid chromatography system was Waters 717 Plus autosampler (Waters, Milford, MA) with Waters 626 LC system (Waters) and for multiple reaction monitoring Quattro Micro triple quadrupole mass spectrometer (Waters/Micromass, UK). Synergi Fusion-RP 80A (250 mm × 2 mm, 4 μm, Phenomenex, Torrance, CA, USA) was used in separation. 0.1% formic acid in H_2_O was used as buffer A and 0.1% formic acid in methanol as buffer B. Flow rate was 170 μl/min. Samples were run on stepwise gradient with steps from 100% buffer A (0–3 min), 0% buffer B to 50% buffer B (3–3.5 min) and 50% buffer B (3.5-6 min). Mass spectrometer was run on negative mode with capillary voltage set to 3 kV and source temperature to 120°C. MRM transitions were m/z 308.0 → 86.9 for Neu5Ac with collision energy 17 V; m/z 220.0 → 58.8 for ManNAc with collision energy 16 V and m/z 185.0 → 92.0 for labeled fructose with collision energy 9 V. The MRM data was analyzed and quantified with Quanlynx software (Waters). Quantification data was exported to Excel (Microsoft, Redmond, WA, USA) and quantification values were normalized based on labeled fructose internal standard. To correct for cell proliferation the final values were also normalized by cell proliferation factor (= time point cell count / zero-hour cell count) obtained from hemocytometer calculations.

### LC-MS^E^ analysis and protein quantification

Peptides were suspended to 80 μl of 3% acetonitrile in 0.1% formic acid. 20 μl of Hi3 Ecoli standard loading control (Waters) was added to samples to final concentration of 10 fmol/μl. 5 μl was used in MS analysis with three technical replicates run of all samples. The used LC instrumentation was nanoACQUITY UPLC (Waters) coupled to Synapt G2-S HDMS mass spectrometer (Waters). For peptide trapping we used nanoACQUITY UPLC Symmetry C18 trap column (180 μm × 20 mm, 5 μm, Waters) and for analytical separation nanoACQUITY UPLC BEH130 C18 (75 μm × 150 mm, 1.7 μm, Waters) column. Mobile solvents were 0.1% formic acid in H_2_O as buffer A and 0.1% formic acid in acetonitrile as buffer B. Gradient was run with 350 nl/min flow rate at 30°C. Liquid chromatography separation was performed using linear gradient from initial buffer B concentration of 3% to 30% over 140 minutes. Data was collected using MS^E^ data acquisition method with IMS separation for 140 minutes. 100 fM Glu-fibrinopeptide B was used as lockmass compound. Fragmentation data was collected for one second at low energy using 4 V and for elevated energy using ramp from 20 V to 42 V. IMS wave velocity was set to 900 m/s. Raw data was imported to Waters PLGS 3.0 software (Waters) and processed using low energy threshold of 100 counts and elevated energy threshold of 25 counts. Intensity Threshold was set to 750 counts. Peptide identifications were done using UniProt Human reference database (dated 1.11.2012). Digest reagent was set to trypsin with one allowed missed cleavage. Carbamidomethylated cysteine was set as fixed modification and oxidation of methionine as variable modification.

Protein quantification was performed by PLGS Expression-E software. Autonormalization option was selected as normalization method. Ratio cut-off of 1.3 (approximately six times the average intensity error) was used as significant change in protein abundance. Up-or downregulation probability of ≥95% and good peptide identification (Ok- designation in PLGS) were required for protein quantification. Only those proteins with good quantification in one technical and at least in two biological replicates were considered as significant. The data was manually curated to search for proteins identified with same peptides which may be the case for highly similar protein isoforms. Only those protein isoforms were accepted for quantification where there was at least one unique peptide hit to the respective isoform.

### Functional annotations

All BiNGO analysis was performed using list of all identified 1802 proteins as a background set eliminating the bias that may rise from the sample preparation or MS detection. To gain a broader view of the Gene Ontology processes we used GO Slim as the BiNGO enrichment ontology. DAVID analysis [43,44] was performed using the same background set of 1802 proteins. Annotation categories were Protein Information Resource keywords, KEGG and Reactome pathways, PANTHER terms and Gene Ontology Biological Process, Molecular Function and Cellular Component categories. Ingenuity Pathway Analysis was done using IPA Core Analysis workflow (Ingenuity Systems, [45]) with default settings.

### Western blot validation

Equal amount of sample from each biological replicate was loaded to Criterion TGX Stain Free precast 10%-20% gradient gel (Bio-Rad, Richmond, CA, USA) and blotted to PVDF membrane (Hybond-LFP, GE Healthcare, UK) using Fast Semi-dry blotter (Thermo/Pierce). Membranes were blocked overnight at 4°C in 2% bovine serum albumin in PBS with 1% Tween 20 in gentle rocking platform. Primary antibody dilutions were done in PBS with 1% Tween 20 using dilutions 1:500 for ATIC antibody (ab33520, Abcam, Cambridge, MA, USA), 1:1000 for RAB5 antibody (ab18211, Abcam), 1:500 for NME2 antibody (MCA4950Z, Abd Serotec, UK) and 1:2000 antibody dilution of loading control beta Actin (ab8227, Abcam). Membranes were incubated in primary antibody for 90 minutes at room temperature with gentle rocking. After primary antibody incubation membranes were washed three times briefly with PBS with 1% Tween 20 then three 10 minute washes with same buffer in gentle rocking. Secondary antibodies were prepared 1:1000 in PBS with 1% Tween 20. HRP conjugated polyclonal rabbit anti-mouse antibody (DAKO, Denmark) was used for ATIC and NME2 and HRP conjugated polyclonal goat anti-rabbit antibody (DAKO) for RAB5 and beta Actin. Washes were performed as with primary antibodies. The detection was done with ECL Plus Western Blot detection system (GE Healthcare) and membranes were scanned with Typhoon 9400 variable mode imager (GE Healthcare). WB quantification was done using ImageQuant TL (GE Healthcare) software and Excel (Microsoft).

## Abbreviations

MS: Mass spectrometry; MRM: Multiple reaction monitoring; LC: Liquid chromatography; UPLC: Ultraperformance liquid chromatography; IMS: Ion mobility separation; EMRT cluster: Exact mass and retention time cluster; Neu5Ac: N-Acetylneuraminic acid; CMP-Neu5Ac: Cytidine-5′-monophospho-N-Acetylneuraminic acid; ManNAc: N-Acetylmannosamine; IPA: Ingenuity pathway analysis; WB: Western blot; SAM: S-adenosylmethionine.

## Competing interests

The authors declare that they have no competing interests.

## Authors’ contributions

VP carried out the experimental steps and wrote the paper. VP and SJ performed the mass spectrometric analysis of proteins. VP and NT carried out the metabolite MS analysis. Analysis design and manuscript revision was done by VP, NT, SJ and RR. All authors read and approved the manuscript.

## Supplementary Material

Additional file 1 **MS data quality evaluation.** Data quality evaluation and technical reproducibility of PLGS Expression-E quantifications of biological replicates. Click here for file

Additional file 2 **All MS identification and quantifications.** Mass spectrometry quantification results of all proteins identified in three biological replicates. Click here for file

Additional file 3 **Significantly changed proteins.** Ratio is represented as an average of three biological replicate induced/control sample ratios. Click here for file

Additional file 4 **Functional enrichments.** Results from functional enrichments of significantly changed proteins in BiNGO, DAVID and IPA categories. Click here for file
