# Supplementary material for: Label-free mass spectrometry proteome quantification of human embryonic kidney cells following 24 hours of sialic acid overproduction
Source: Proteome Sci. 2013 Aug 1;11:38. doi: 10.1186/1477-5956-11-38 (PMC3750590; doi:10.1186/1477-5956-11-38)
Supplement: Additional file 1 — MS data quality evaluation. Data quality evaluation and technical reproducibility of PLGS Expression-E quantifications of biological replicates. [file 1477-5956-11-38-S1.docx]

**Additional file 1. MS quantification, data quality evaluation**

**A: EMRT cluster intensity coefficient of variation between three technical replicate runs:**


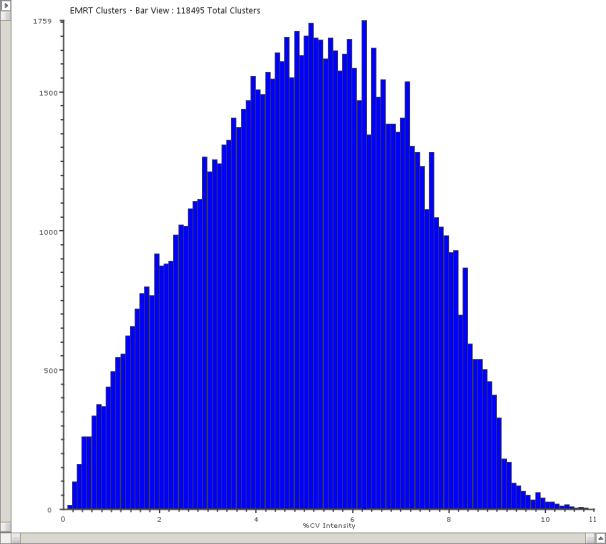

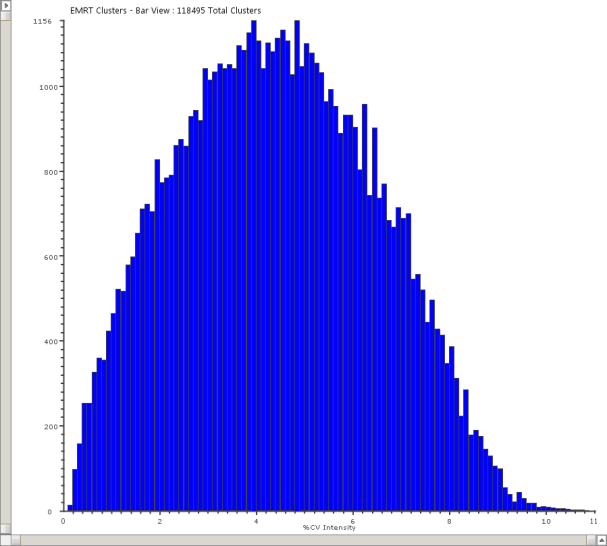


Biological replicate 1. Control Biological replicate 1. Induced


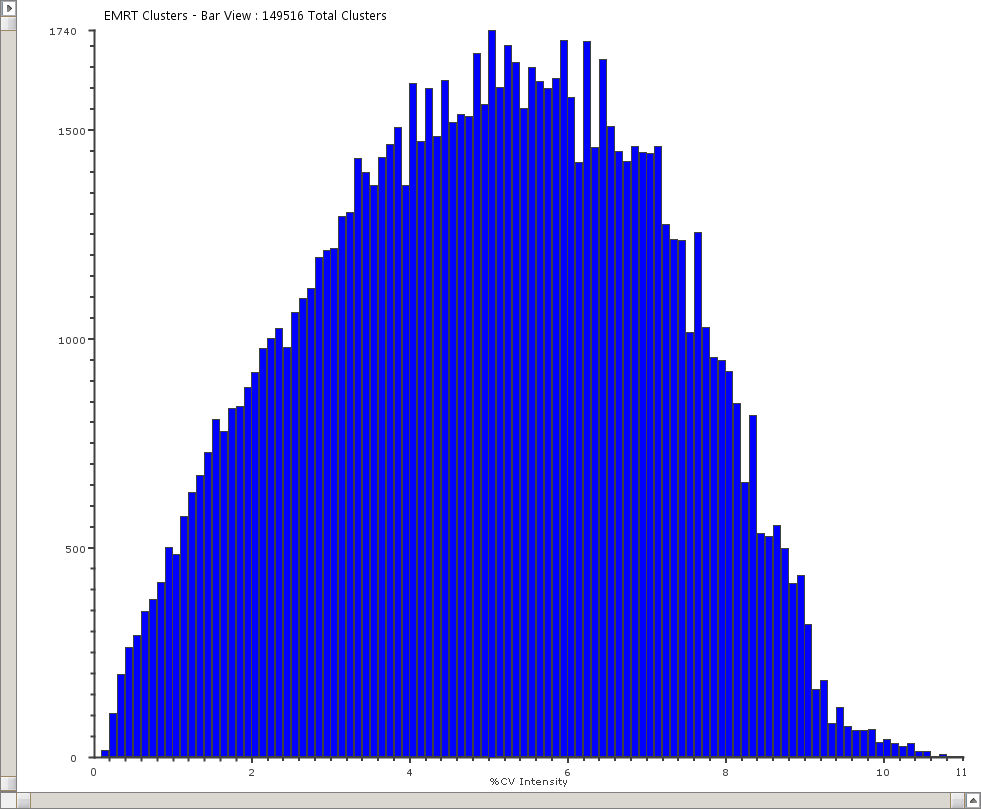

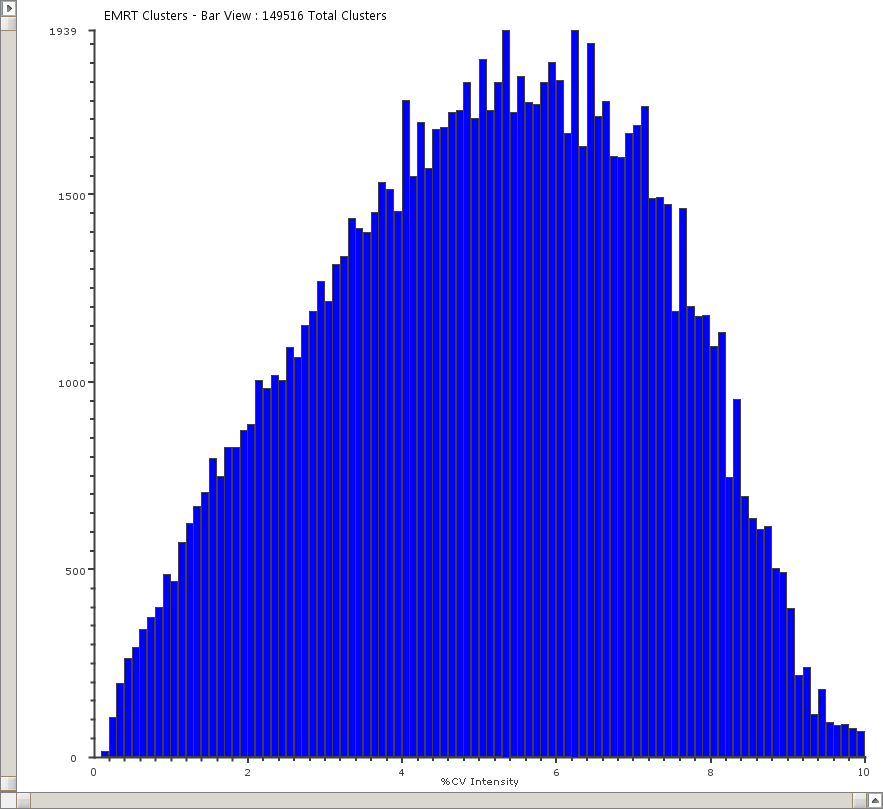


Biological replicate 2. Control Biological replicate 2. Induced


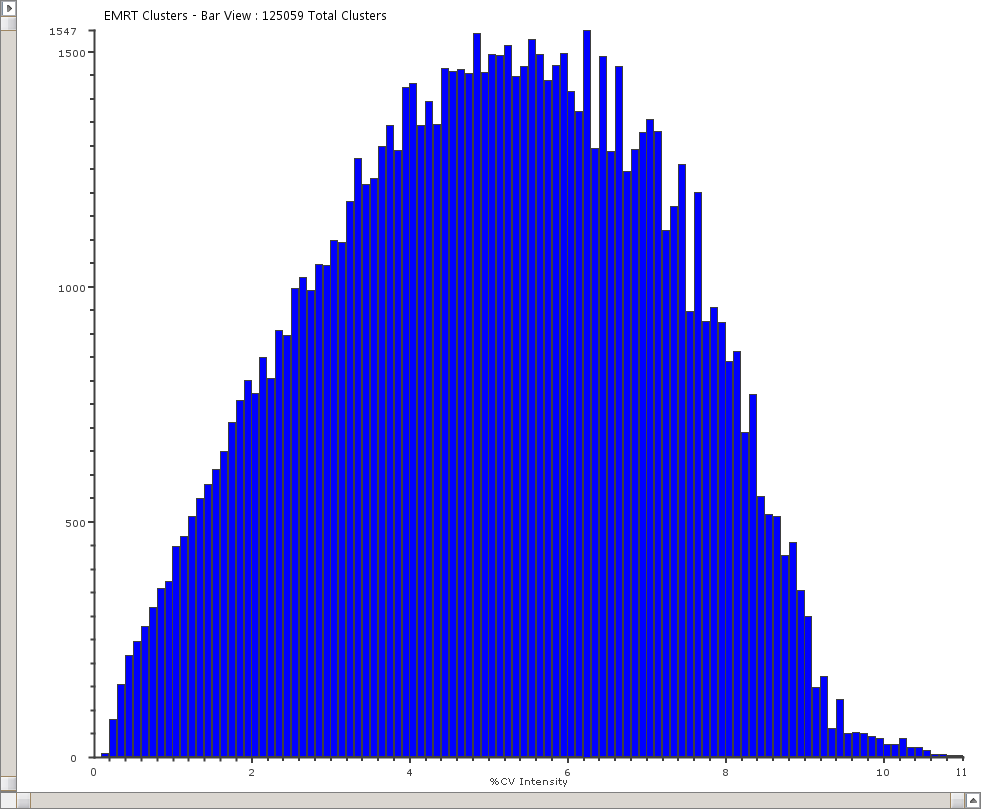

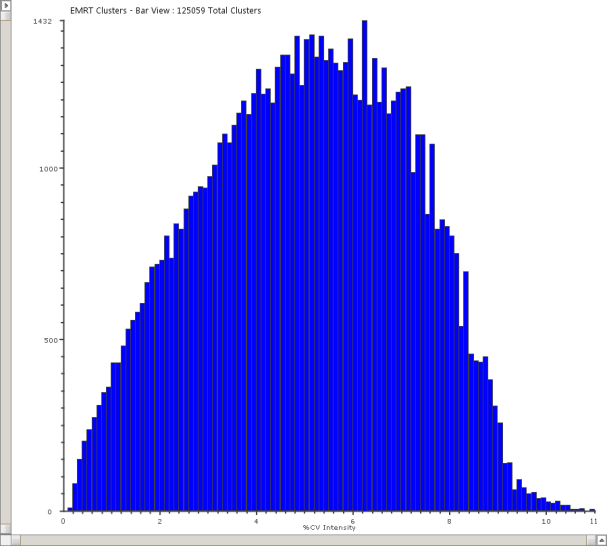


Biological replicate 3. Control Biological replicate 3. Induced

**B: EMRT cluster retention time coefficient of variation between three technical replicate runs:**


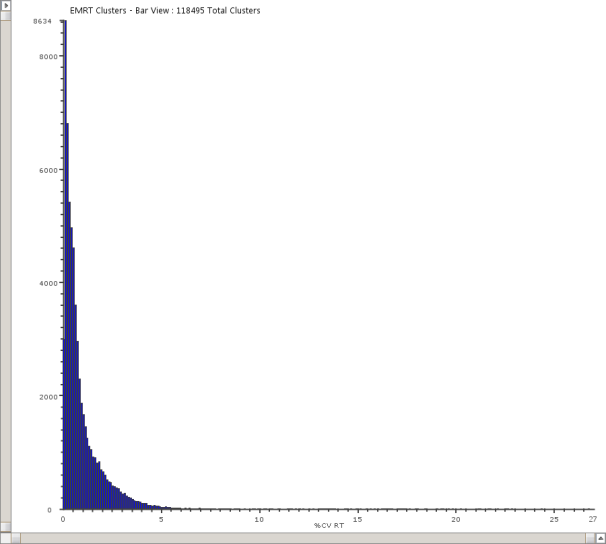

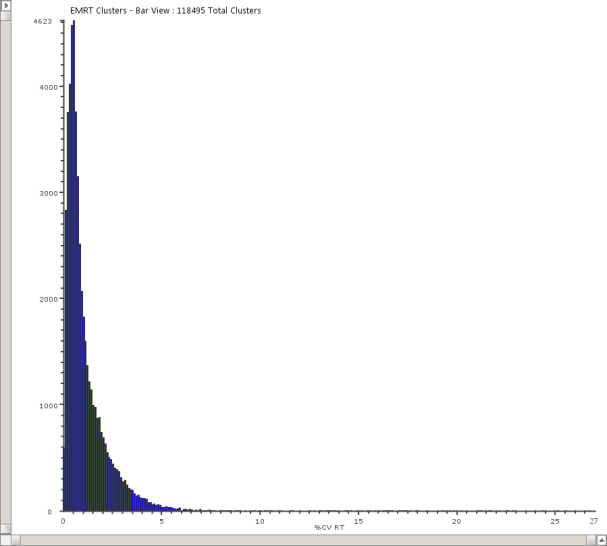


Biological replicate 1. Control Biological replicate 1. Induced


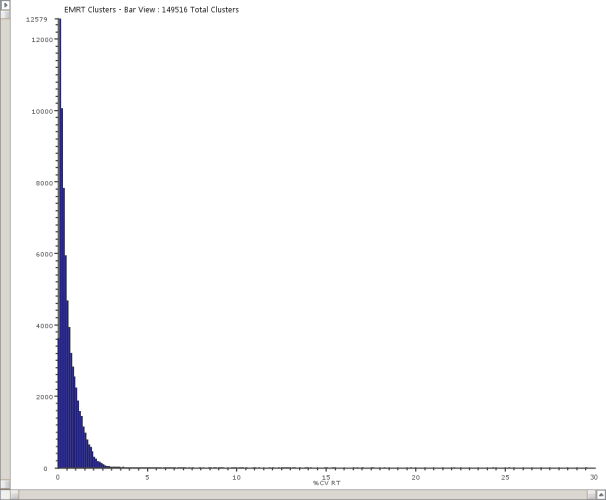

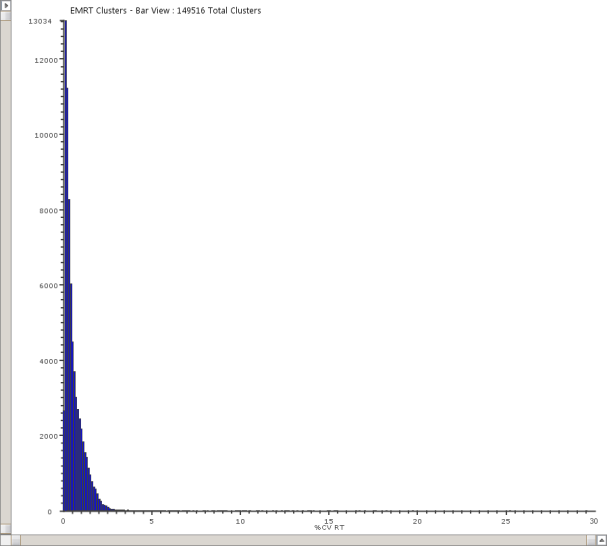


Biological replicate 2. Control Biological replicate 2. Induced


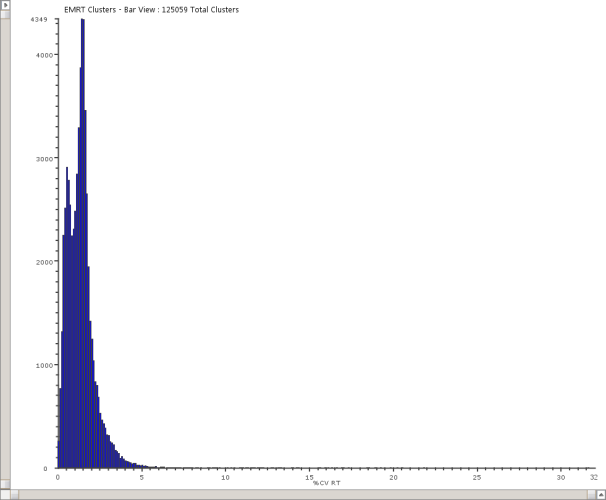

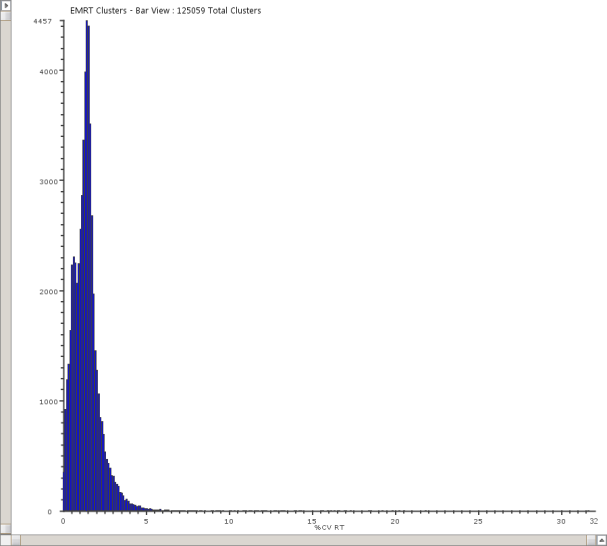


Biological replicate 3. Control Biological replicate 3. Induced

**C: EMRT cluster and protein replication rate between three technical replicate runs:**


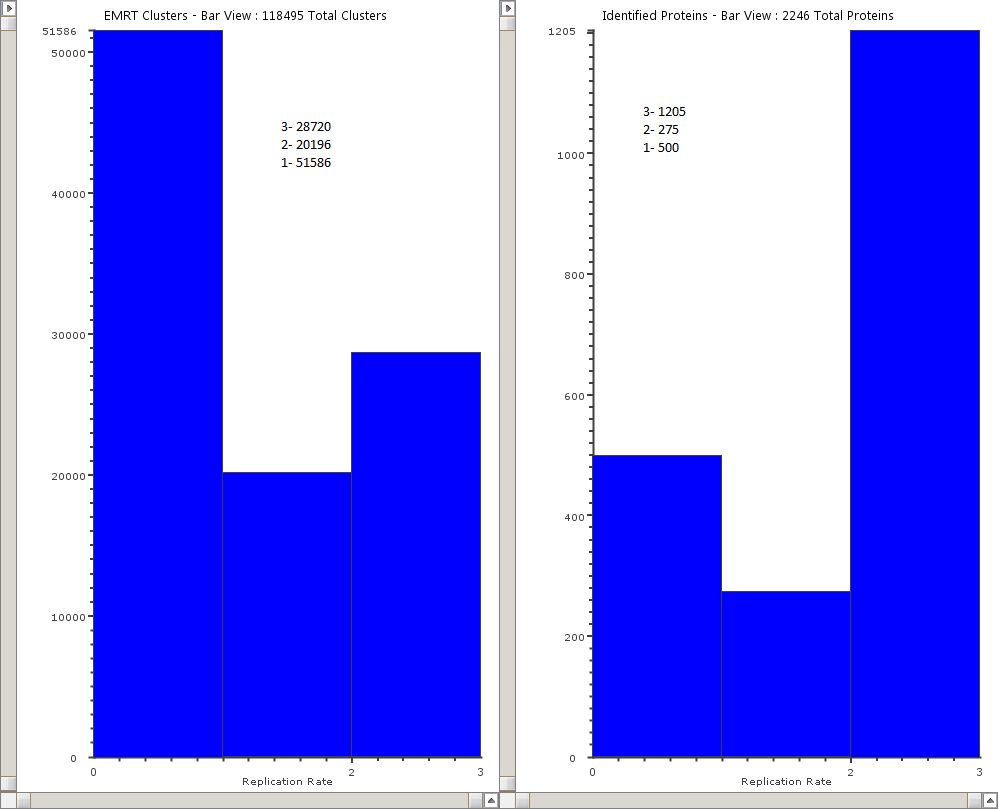

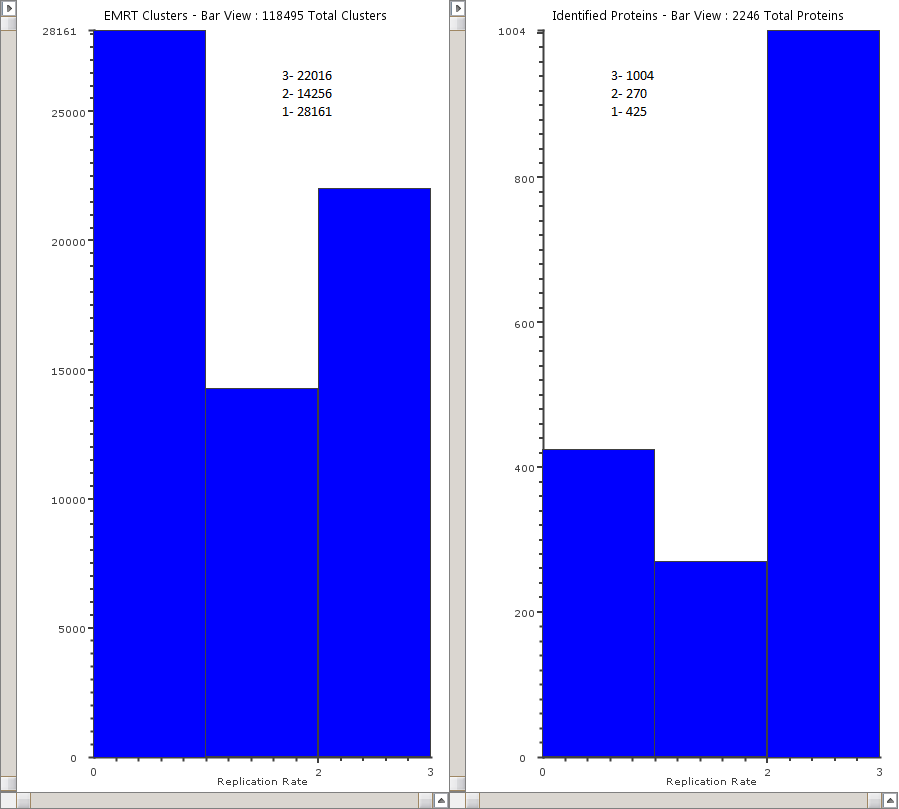


Biological replicate 1. Control Biological replicate 1. Induced


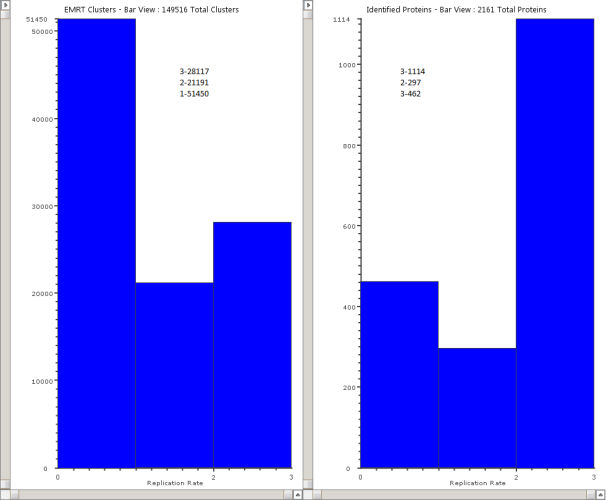

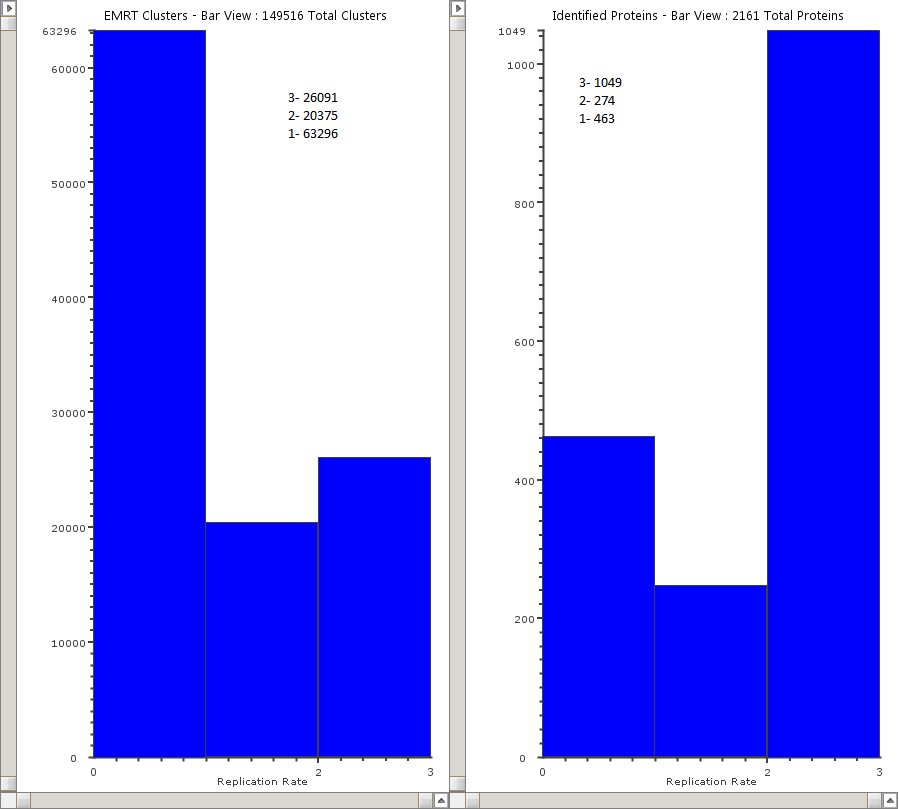


Biological replicate 2. Control Biological replicate 2. Induced


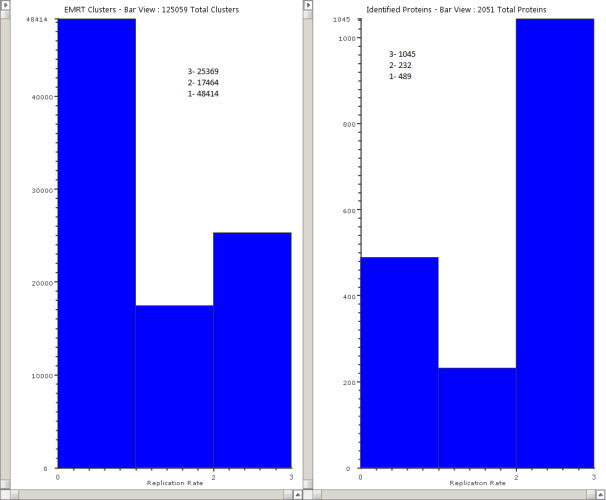

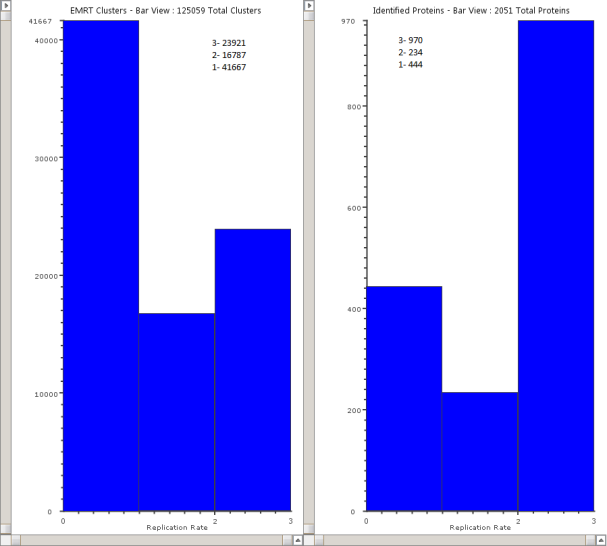


Biological replicate 3. Control Biological replicate 3. Induced

**D: EMRT cluster mass relative standard deviation between three technical replicate runs:**


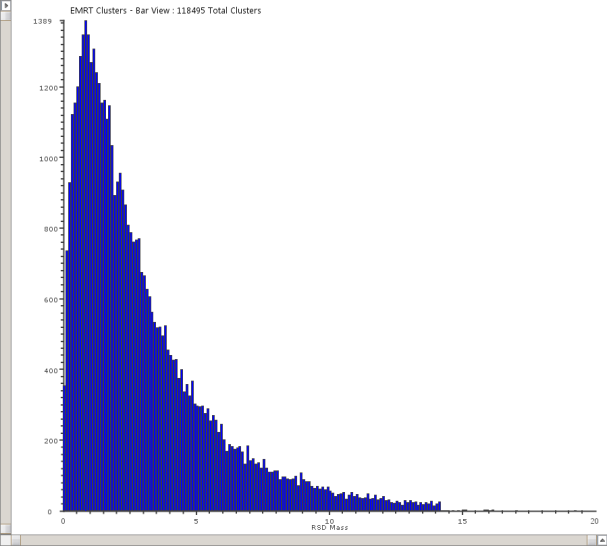

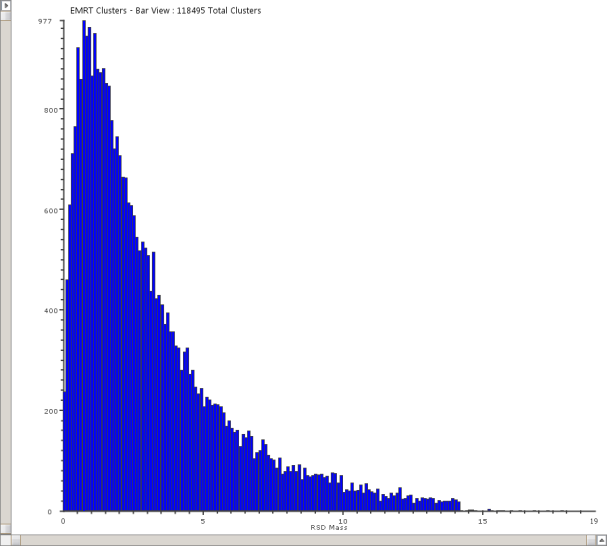


Biological replicate 1. Control Biological replicate 1. Induced


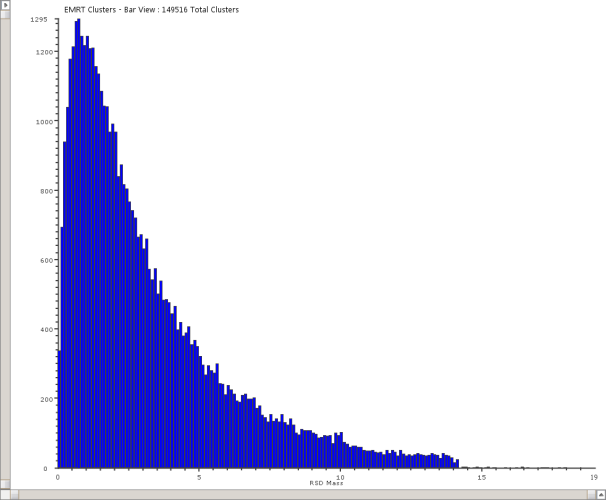

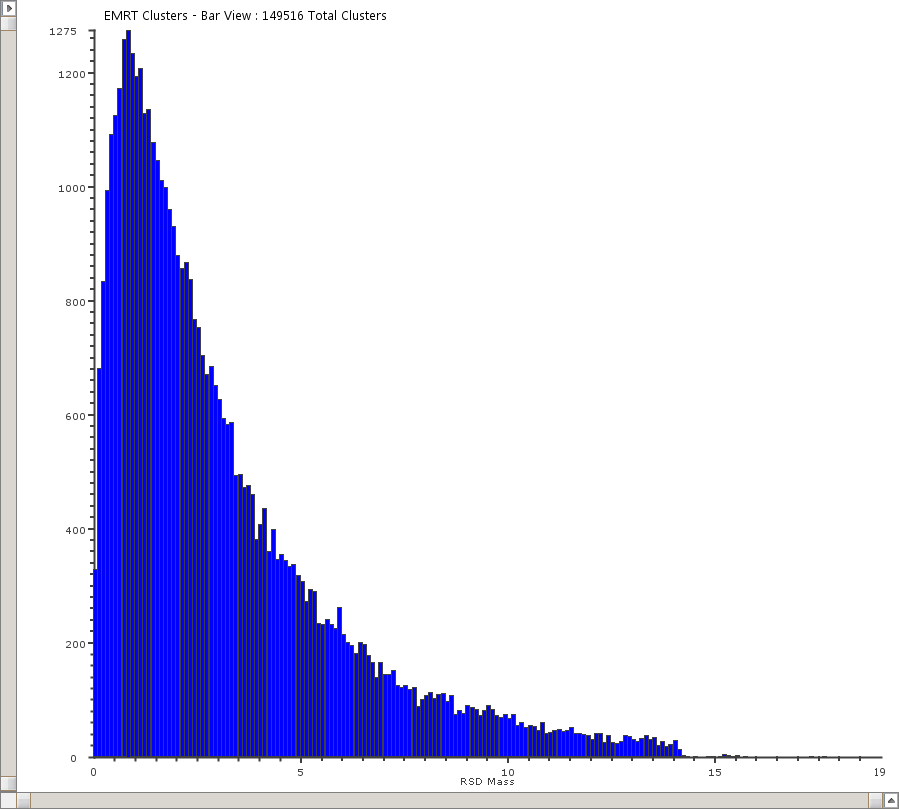


Biological replicate 2. Control Biological replicate 2. Induced


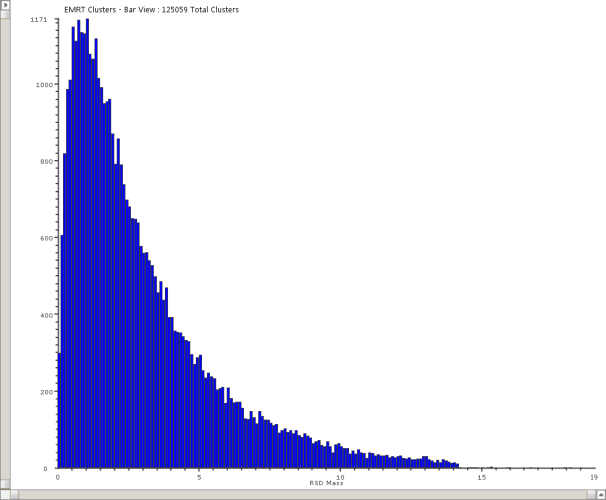

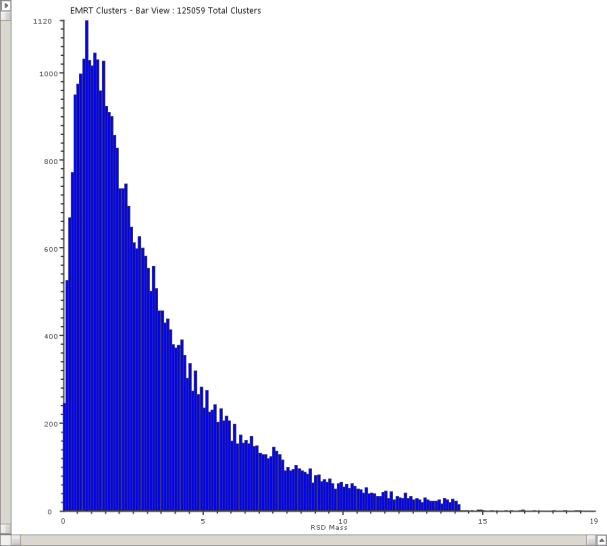


Biological replicate 3. Control Biological replicate 3. Induced

**E: Representative scatter plots of two technical replicate runs:**


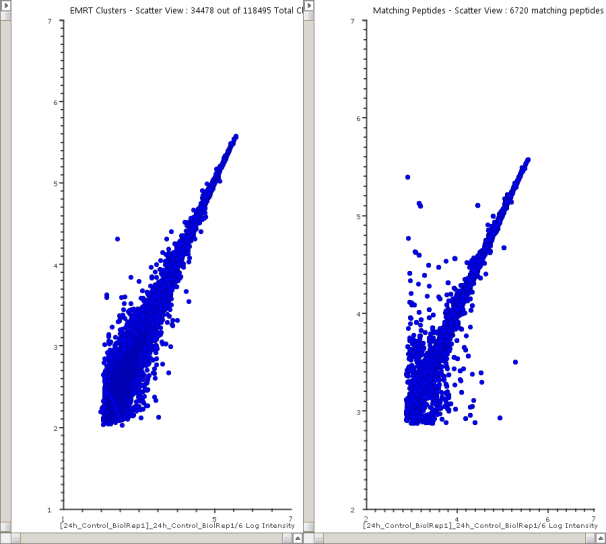

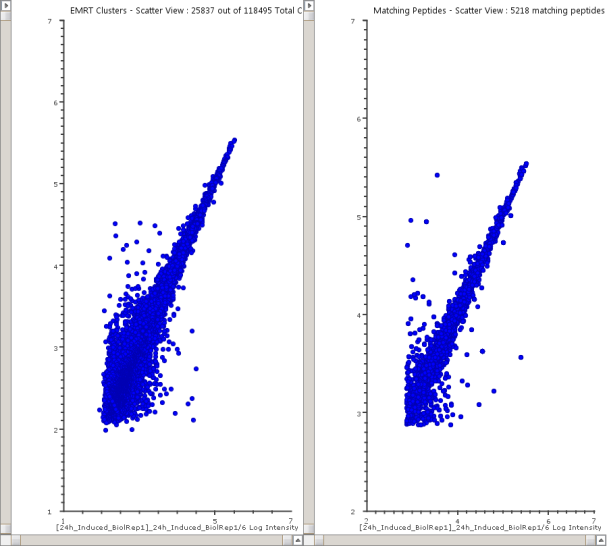


Biological replicate 1. Control Biological replicate 1. Induced


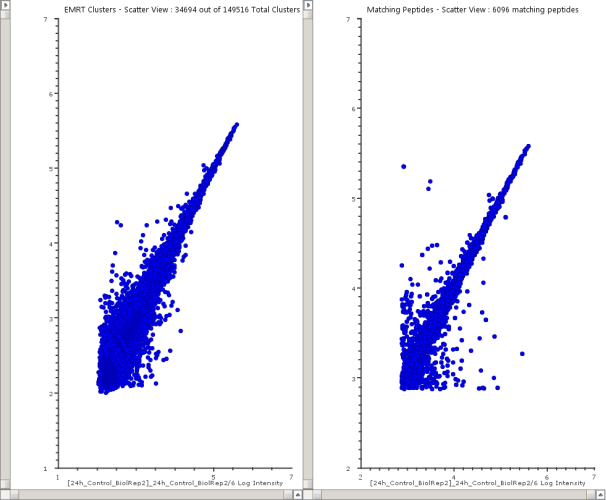

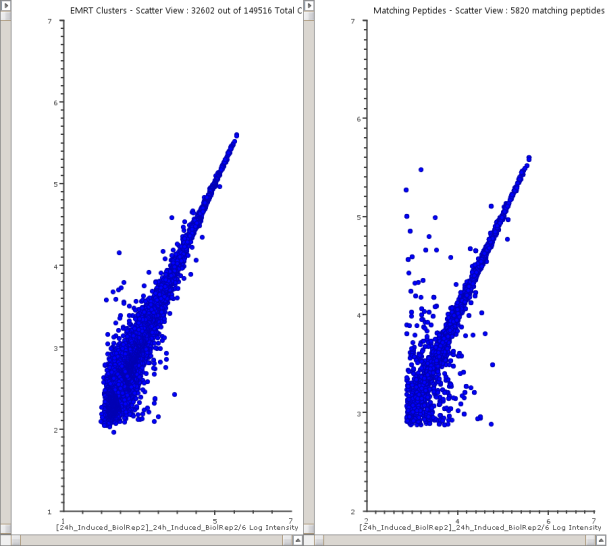


Biological replicate 2. Control Biological replicate 2. Induced


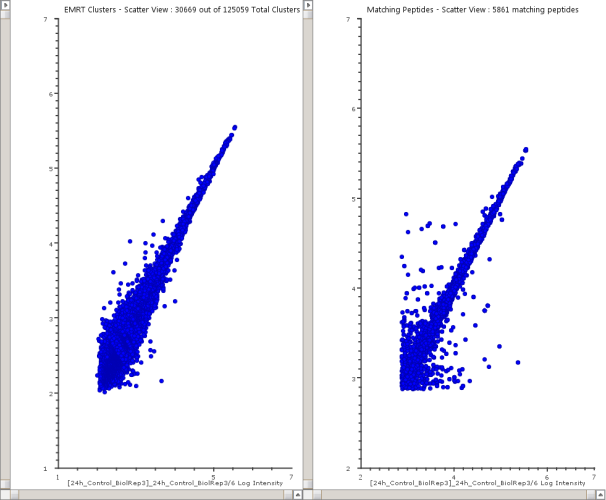

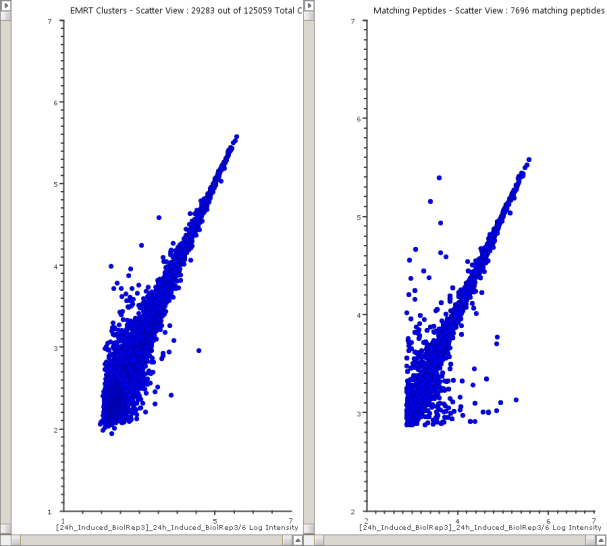


Biological replicate 3. Control Biological replicate 3. Induced
