# Supplementary material for: Label-free mass spectrometry proteome quantification of human embryonic kidney cells following 24 hours of sialic acid overproduction
Source: Proteome Sci. 2013 Aug 1;11:38. doi: 10.1186/1477-5956-11-38 (PMC3750590; doi:10.1186/1477-5956-11-38)
Supplement: Additional file 3 — Significantly changed proteins. Ratio is represented as an average of three biological replicate induced/control sample ratios. [file 1477-5956-11-38-S3.doc]

## Table 1 - Significantly changed proteins

| **Significantly changed proteins** |  |  |  |  |
| --- | --- | --- | --- | --- |
| **Name** | **Official name** | **Swiss-Prot Accession** | **Induced/Control ratio ± standard deviation** | **Functional categories** |
| Cyclin-dependent kinase 18 | CDK18 | [Swiss-Prot:Q07002] | 0.07 ± 0.02 | Signaling |
| RNA-binding motif protein, X-linked-like-3 | RBMXL3 | [Swiss-Prot:Q8N7X1] | 0.26 ± 0.2 |  |
| Nucleosome assembly protein 1-like 1 | NAP1L1 | [Swiss-Prot:P55209] | 0.28 ± 0.03 |  |
| GTP-binding protein SAR1b | SAR1B | [Swiss-Prot:Q9Y6B6] | 0.29 ± 0.11 | Protein transport, Golgi, Small GTPase |
| GTP-binding protein SAR1a | SAR1A | [Swiss-Prot:Q9NR31] | 0.31 ± 0.09 | Signaling, Protein transport, Golgi, Small GTPase |
| ADP-ribosylation factor 4 | ARF4 | [Swiss-Prot:P18085] | 0.34 ± 0.07 | Plasma membrane, Signaling, Protein transport, Golgi, Small GTPase |
| ADP-ribosylation factor 5 | ARF5 | [Swiss-Prot:P84085] | 0.36 ± 0.07 | Plasma membrane, Signaling, Protein transport, Golgi, Small GTPase |
| Complement component 1 Q subcomponent-binding protein, mitochondrial | C1QBP | [Swiss-Prot:Q07021] | 0.36 ± 0.13 | Plasma membrane |
| Eukaryotic translation initiation factor 5A-2 | EIF5A2 | [Swiss-Prot:Q9GZV4] | 0.36 ± 0.14 | Plasma membrane, Protein transport |
| Neudesin | NENF | [Swiss-Prot:Q9UMX5] | 0.37 ± 0.05 |  |
| Eukaryotic translation initiation factor 5A-1 | EIF5A | [Swiss-Prot:P63241] | 0.38 ± 0.08 | Plasma membrane, Protein transport |
| THO complex subunit 4 | ALYREF | [Swiss-Prot:Q86V81] | 0.4 ± 0.1 |  |
| F-actin-capping protein subunit alpha-2 | CAPZA2 | [Swiss-Prot:P47755] | 0.42 ± 0.15 |  |
| Hemoglobin subunit alpha | HBA1 | [Swiss-Prot:P69905] | 0.42 ± 0.21 |  |
| LETM1 and EF-hand domain-containing protein 1, mitochondrial | LETM1 | [Swiss-Prot:O95202] | 0.42 ± 0.43 |  |
| Cystatin-B | CSTB | [Swiss-Prot:P04080] | 0.43 ± 0.13 |  |
| 60S ribosomal protein L24 | RPL24 | [Swiss-Prot:P83731] | 0.43 ± 0.14 | Signaling |
| 60S acidic ribosomal protein P2 | RPLP2 | [Swiss-Prot:P05387] | 0.45 ± 0.07 |  |
| Alpha-actinin-3 | ACTN3 | [Swiss-Prot:Q08043] | 0.46 ± 0.06 | Plasma membrane, Adherens junction |
| Transaldolase | TALDO1 | [Swiss-Prot:P37837] | 0.46 ± 0.28 |  |
| 14-3-3 protein sigma | SFN | [Swiss-Prot:P31947] | 0.47 ± 0.34 | Signaling |
| ATPase family AAA domain-containing protein 3C | ATAD3C | [Swiss-Prot:Q5T2N8] | 0.48 ± 0.12 |  |
| Eukaryotic translation initiation factor 3 subunit M | EIF3M | [Swiss-Prot:Q7L2H7] | 0.49 ± 0.08 |  |
| Tropomyosin alpha-1 chain | TPM1 | [Swiss-Prot:P09493] | 0.49 ± 0.15 | Plasma membrane |
| Splicing factor 3A subunit 3 | SF3A3 | [Swiss-Prot:Q12874] | 0.5 ± 0.04 |  |
| NHP2-like protein 1 | NHP2L1 | [Swiss-Prot:P55769] | 0.5 ± 0.11 |  |
| Polyadenylate-binding protein 3 | PABPC3 | [Swiss-Prot:Q9H361] | 0.5 ± 0.26 |  |
| Methylosome protein 50 | WDR77 | [Swiss-Prot:Q9BQA1] | 0.5 ± 0.32 |  |
| Prefoldin subunit 2 | PFDN2 | [Swiss-Prot:Q9UHV9] | 0.51 ± 0.07 |  |
| Ran-specific GTPase-activating protein | RANBP1 | [Swiss-Prot:P43487] | 0.51 ± 0.23 | Signaling |
| Peptidyl-prolyl cis-trans isomerase A | PPIA | [Swiss-Prot:P62937] | 0.52 ± 0.1 |  |
| Ubiquilin-4 | UBQLN4 | [Swiss-Prot:Q9NRR5] | 0.53 ± 0.1 |  |
| Serine--tRNA ligase, cytoplasmic | SARS | [Swiss-Prot:P49591] | 0.53 ± 0.21 |  |
| Programmed cell death protein 5 | PDCD5 | [Swiss-Prot:O14737] | 0.53 ± 0.29 |  |
| Microtubule-associated protein RP/EB family member 1 | MAPRE1 | [Swiss-Prot:Q15691] | 0.54 ± 0.02 | Plasma membrane, Golgi, Adherens junction |
| ATP-dependent RNA helicase DDX50 | DDX50 | [Swiss-Prot:Q9BQ39] | 0.54 ± 0.03 |  |
| Glycogen phosphorylase, brain form | PYGB | [Swiss-Prot:P11216] | 0.54 ± 0.08 |  |
| Galectin-3-binding protein | LGALS3BP | [Swiss-Prot:Q08380] | 0.54 ± 0.13 | Signaling |
| Tubulin beta-4A chain | TUBB4A | [Swiss-Prot:P04350] | 0.54 ± 0.15 | Adherens junction |
| Signal recognition particle 9 kDa protein | SRP9 | [Swiss-Prot:P49458] | 0.54 ± 0.18 | Protein transport |
| Putative tubulin beta chain-like protein ENSP00000290377 |  | [Swiss-Prot:A6NKZ8] | 0.54 ± 0.2 |  |
| Nascent polypeptide-associated complex subunit alpha | NACA | [Swiss-Prot:Q13765] | 0.55 ± 0.01 | Protein transport |
| Nucleoside diphosphate kinase B | NME2 | [Swiss-Prot:P22392] | 0.55 ± 0.03 | Pyrimidine biosynthesis |
| Eukaryotic translation initiation factor 3 subunit F | EIF3F | [Swiss-Prot:O00303] | 0.55 ± 0.08 |  |
| Nucleoside diphosphate kinase A | NME1 | [Swiss-Prot:P15531] | 0.55 ± 0.09 | Adherens junction, Pyrimidine biosynthesis |
| Alpha-actinin-2 | ACTN2 | [Swiss-Prot:P35609] | 0.56 ± 0.15 | Plasma membrane, Adherens junction |
| Polyadenylate-binding protein 4 | PABPC4 | [Swiss-Prot:Q13310] | 0.56 ± 0.18 |  |
| Glycine cleavage system H protein, mitochondrial | GCSH | [Swiss-Prot:P23434] | 0.57 ± 0.03 |  |
| Asparagine--tRNA ligase, cytoplasmic | NARS | [Swiss-Prot:O43776] | 0.57 ± 0.1 |  |
| Interleukin enhancer-binding factor 2 | ILF2 | [Swiss-Prot:Q12905] | 0.57 ± 0.16 |  |
| 60S ribosomal protein L30 | RPL30 | [Swiss-Prot:P62888] | 0.58 ± 0.13 |  |
| Mitochondrial import inner membrane translocase subunit Tim13 | TIMM13 | [Swiss-Prot:Q9Y5L4] | 0.59 ± 0.04 | Protein transport |
| 60S ribosomal protein L7a | RPL7A | [Swiss-Prot:P62424] | 0.59 ± 0.06 |  |
| 26S protease regulatory subunit 10B | PSMC6 | [Swiss-Prot:P62333] | 0.59 ± 0.07 |  |
| U6 snRNA-associated Sm-like protein LSm2 | LSM2 | [Swiss-Prot:Q9Y333] | 0.59 ± 0.12 |  |
| Tubulin beta-1 chain | TUBB1 | [Swiss-Prot:Q9H4B7] | 0.59 ± 0.15 | Adherens junction |
| Cytochrome c | CYCS | [Swiss-Prot:P99999] | 0.6 ± 0.05 |  |
| S-adenosylmethionine synthase isoform type-2 | MAT2A | [Swiss-Prot:P31153] | 0.6 ± 0.1 |  |
| Tropomyosin beta chain | TPM2 | [Swiss-Prot:P07951] | 0.61 ± 0.19 |  |
| 60S ribosomal protein L11 | RPL11 | [Swiss-Prot:P62913] | 0.62 ± 0.03 | Protein transport |
| Prostaglandin E synthase 3 | PTGES3 | [Swiss-Prot:Q15185] | 0.62 ± 0.05 | Signaling |
| Annexin A6 | ANXA6 | [Swiss-Prot:P08133] | 0.62 ± 0.06 | Plasma membrane |
| Calreticulin | CALR | [Swiss-Prot:P27797] | 0.62 ± 0.09 | Plasma membrane, Signaling, Protein transport |
| Cytochrome c oxidase subunit 2 | MT-CO2 | [Swiss-Prot:P00403] | 0.63 ± 0.01 |  |
| DnaJ homolog subfamily A member 1 | DNAJA1 | [Swiss-Prot:P31689] | 0.63 ± 0.01 | Signaling |
| Amidophosphoribosyltransferase | PPAT | [Swiss-Prot:Q06203] | 0.63 ± 0.04 | Purine biosynthesis |
| Ras GTPase-activating protein-binding protein 1 | G3BP1 | [Swiss-Prot:Q13283] | 0.63 ± 0.11 | Plasma membrane, Signaling |
| Proteasome activator complex subunit 3 | PSME3 | [Swiss-Prot:P61289] | 0.63 ± 0.12 |  |
| Bifunctional purine biosynthesis protein PURH | ATIC | [Swiss-Prot:P31939] | 0.63 ± 0.16 | Purine biosynthesis |
| Gamma-glutamylcyclotransferase | GGCT | [Swiss-Prot:O75223] | 0.64 ± 0.04 |  |
| ADP/ATP translocase 2 | SLC25A5 | [Swiss-Prot:P05141] | 0.64 ± 0.07 | Plasma membrane |
| Endoplasmic reticulum resident protein 29 | ERP29 | [Swiss-Prot:P30040] | 0.64 ± 0.09 | Protein transport, |
| Methionine adenosyltransferase 2 subunit beta | MAT2B | [Swiss-Prot:Q9NZL9] | 0.64 ± 0.12 |  |
| Protein disulfide-isomerase A6 | PDIA6 | [Swiss-Prot:Q15084] | 0.65 ± 0.06 | Plasma membrane |
| Cyclin-dependent kinase inhibitor 2A, isoforms 1/2/3 | CDKN2A | [Swiss-Prot:P42771] | 0.65 ± 0.1 | Signaling |
| Guanine nucleotide-binding protein G(k) subunit alpha | GNAI3 | [Swiss-Prot:P08754] | 0.65 ± 0.11 | Plasma membrane, Signaling, Golgi |
| Tropomyosin alpha-4 chain | TPM4 | [Swiss-Prot:P67936] | 0.65 ± 0.15 |  |
| X-ray repair cross-complementing protein 6 | XRCC6 | [Swiss-Prot:P12956] | 0.66 ± 0.02 |  |
| Dynamin-2 | DNM2 | [Swiss-Prot:P50570] | 0.66 ± 0.07 | Plasma membrane, Signaling, Protein transport, Adherens junction |
| Guanine nucleotide-binding protein G(i) subunit alpha-1 | GNAI1 | [Swiss-Prot:P63096] | 0.66 ± 0.1 | Plasma membrane, Signaling |
| Ras-related protein Rab-6B | RAB6B | [Swiss-Prot:Q9NRW1] | 0.66 ± 0.12 | Signaling, Protein transport, Golgi, Small GTPase |
| Destrin | DSTN | [Swiss-Prot:P60981] | 0.67 ± 0.01 |  |
| Aconitate hydratase, mitochondrial | ACO2 | [Swiss-Prot:Q99798] | 0.67 ± 0.01 |  |
| Ubiquitin carboxyl-terminal hydrolase 14 | USP14 | [Swiss-Prot:P54578] | 0.67 ± 0.02 | Plasma membrane |
| Ras-related protein Rab-5C | RAB5C | [Swiss-Prot:P51148] | 0.67 ± 0.04 | Plasma membrane, Signaling, Protein transport, Small GTPase, Adherens junction |
| Crk-like protein | CRKL | [Swiss-Prot:P46109] | 0.67 ± 0.05 | Signaling |
| Ras-related protein Rab-5A | RAB5A | [Swiss-Prot:P20339] | 0.67 ± 0.08 | Plasma membrane, Signaling, Protein transport, Small GTPase, Adherens junction |
| Chloride intracellular channel protein 1 | CLIC1 | [Swiss-Prot:O00299] | 0.68 ± 0.07 | Plasma membrane, Signaling |
| Coiled-coil-helix-coiled-coil-helix domain-containing protein 2, mitochondrial | CHCHD2 | [Swiss-Prot:Q9Y6H1] | 0.69 ± 0.01 |  |
| Putative histone H2B type 2-C | HIST2H2BC | [Swiss-Prot:Q6DN03] | 0.69 ± 0.02 |  |
| Carbonic anhydrase 2 | CA2 | [Swiss-Prot:P00918] | 0.69 ± 0.07 | Plasma membrane |
| Glia maturation factor beta | GMFB | [Swiss-Prot:P60983] | 0.7 ± 0 | Signaling |
| 40S ribosomal protein S30 | FAU | [Swiss-Prot:P62861] | 0.7 ± 0.03 |  |
| Ubiquitin-conjugating enzyme E2 K | UBE2K | [Swiss-Prot:P61086] | 0.7 ± 0.04 |  |
| U6 snRNA-associated Sm-like protein LSm3 | LSM3 | [Swiss-Prot:P62310] | 0.7 ± 0.06 |  |
| Septin-2 | SEPT2 | [Swiss-Prot:Q15019] | 0.71 ± 0.01 | Plasma membrane |
| Adenylosuccinate synthetase isozyme 2 | ADSS | [Swiss-Prot:P30520] | 0.73 ± 0.01 | Purine biosynthesis |
| S-phase kinase-associated protein 1 | SKP1 | [Swiss-Prot:P63208] | 0.73 ± 0.02 |  |
| Septin-9 | SEPT9 | [Swiss-Prot:Q9UHD8] | 1.36 ± 0.02 | Plasma membrane |
| Transgelin-3 | TAGLN3 | [Swiss-Prot:Q9UI15] | 1.4 ± 0.01 |  |
| 40S ribosomal protein S3a | RPS3A | [Swiss-Prot:P61247] | 1.45 ± 0.04 |  |
| Myosin regulatory light polypeptide 9 | MYL9 | [Swiss-Prot:P24844] | 1.51 ± 0.03 |  |
| Mitochondrial import receptor subunit TOM6 homolog | TOMM6 | [Swiss-Prot:Q96B49] | 1.63 ± 0.15 | Protein transport |
| ADP/ATP translocase 4 | SLC25A31 | [Swiss-Prot:Q9H0C2] | 1.87 ± 0.39 |  |
| Ras-related protein Rab-13 | RAB13 | [Swiss-Prot:P51153] | 5.18 ± 0.18 | Plasma membrane, Signaling, Protein transport, Golgi, Small GTPase |

Ratio is represented as an average of three biological replicate induced/control sample ratios.
